# Effects of Probiotic Supplementation on the Gut Microbiota and Antibiotic Resistome Development in Preterm Infants

**DOI:** 10.3389/fped.2018.00347

**Published:** 2018-11-16

**Authors:** Eirin Esaiassen, Erik Hjerde, Jorunn Pauline Cavanagh, Tanja Pedersen, Jannicke H. Andresen, Siren I. Rettedal, Ragnhild Støen, Britt Nakstad, Nils P. Willassen, Claus Klingenberg

**Affiliations:** ^1^Paediatric Research Group, Department of Clinical Medicine, UiT The Arctic University of Norway, Tromsø, Norway; ^2^Department of Paediatrics, University Hospital of North Norway, Tromsø, Norway; ^3^Department of Chemistry, Norstruct, UiT The Arctic University of Norway, Tromsø, Norway; ^4^Department of Paediatrics, Haukeland University Hospital, Bergen, Norway; ^5^Department of Neonatal Intensive Care, Oslo University Hospital, Oslo, Norway; ^6^Department of Paediatrics, Stavanger University Hospital, Stavanger, Norway; ^7^Department of Paediatrics, St. Olavs University Hospital, Trondheim, Norway; ^8^Department of Laboratory Medicine, Children‘s and Women‘s Health, University of Science and Technology, Trondheim, Norway; ^9^Department of Paediatric and Adolescents Medicine, Akershus University Hospital, Nordbyhagen, Norway; ^10^Institute of Clinical Medicine, Faculty of Medicine, University of Oslo, Oslo, Norway

**Keywords:** gut microbiota, preterm infant, shotgun metagenome sequencing, taxonomy, bifidobacteria, lactobacilli, colonization resistance

## Abstract

**Objectives:** In 2014 probiotic supplementation (*Lactobacillus acidophilus* and *Bifidobacterium longum* subspecies *infantis;* Infloran^Ⓡ^) was introduced as standard of care to prevent necrotizing enterocolitis (NEC) in extremely preterm infants in Norway. We aimed to evaluate the influence of probiotics and antibiotic therapy on the developing gut microbiota and antibiotic resistome in extremely preterm infants, and to compare with very preterm infants and term infants not given probiotics.

**Study design:** A prospective, observational multicenter study in six tertiary-care neonatal units. We enrolled 76 infants; 31 probiotic-supplemented extremely preterm infants <28 weeks gestation, 35 very preterm infants 28–31 weeks gestation not given probiotics and 10 healthy full-term control infants. Taxonomic composition and collection of antibiotic resistance genes (resistome) in fecal samples, collected at 7 and 28 days and 4 months age, were analyzed using shotgun-metagenome sequencing.

**Results:** Median (IQR) birth weight was 835 (680–945) g and 1,290 (1,150–1,445) g in preterm infants exposed and not exposed to probiotics, respectively. Two extremely preterm infants receiving probiotic developed NEC requiring surgery. At 7 days of age we found higher median relative abundance of *Bifidobacterium* in probiotic supplemented infants (64.7%) compared to non-supplemented preterm infants (0.0%) and term control infants (43.9%). *Lactobacillus* was only detected in small amounts in all groups, but the relative abundance increased up to 4 months. Extremely preterm infants receiving probiotics had also much higher antibiotic exposure, still overall microbial diversity and resistome was not different than in more mature infants at 4 weeks and 4 months.

**Conclusion:** Probiotic supplementation may induce colonization resistance and alleviate harmful effects of antibiotics on the gut microbiota and antibiotic resistome.

**Clinical Trial Registration:** Clinicaltrials.gov: NCT02197468. https://clinicaltrials.gov/ct2/show/NCT02197468

## Introduction

Preterm infants experience unique challenges in establishing their gut microbiota. Cesarean deliveries, extensive antenatal, and neonatal antibiotic exposure, parenteral nutrition and residing for long periods in a neonatal intensive care unit (NICU), may cause unpredictable perturbations of the gut microbiota development (1). Gut microbiota dysbiosis in the first weeks of life is associated with perturbations of the developing immune system (2), and an increased risk of necrotizing enterocolitis (NEC) (3). Probiotic supplementation aims to restore the gut microbiota, and thereby preventing NEC and other complications (4–6). Meta-analyses of randomized and observational trials show that probiotic supplementation, mainly with bifidobacteria and/or lactobacilli, reduce rates of NEC (4, 5, 7, 8). The effects seem to be strain-specific (5) and not all products are efficacious (9). Still, based on recent evidence (4, 10) and expert opinion (11), many NICUs in Europe, Australia, and Canada have implemented routine probiotic-supplementation to preterm infants. Probiotics are infrequently used in preterm infants in the USA (12). Risks of probiotic sepsis and contaminations of probiotic products may explain skepticism (13–16). Some experts recommend waiting for additional studies to confirm the safety and efficacy of an available and reliable product (17). Moreover, there is a paucity of in-depth knowledge on microbiological effects and effective dose of probiotic therapy.

Antibiotics are the most commonly prescribed medications in the NICU (18), and prolonged therapy increases the risk of NEC (19, 20). Antibiotics may influence both the physiological gut microbiota composition and the collection of antibiotic resistance genes (ARGs) in the gut, defined as the gut antibiotic resistome (21, 22). However, there is limited knowledge on how probiotic supplementation and antibiotic therapy influence the gut antibiotic resistome in extremely preterm infants.

In Norway probiotic supplementation was implemented as standard of care for extremely preterm infants in 2014. In a longitudinal multi-center study, using shotgun-metagenomic sequencing, we set out to evaluate the influence of probiotics and antibiotic therapy on the developing gut microbiota and antibiotic resistome in extremely preterm infants supplemented with probiotics. We also compared these results to very preterm infants not supplemented with probiotics and a group of healthy, full-term infants.

## Materials and methods

### Study patients and sampling procedure

We prospectively planned to include two convenient groups of preterm infants from six Norwegian NICUs; one group of extremely preterm infants (gestational age 25–27 weeks) supplemented with probiotics, and one group of very preterm infants (gestational age 28–31 weeks) not supplemented with probiotics. Exclusion criteria were gestation below 25 weeks and/or an early, life threatening condition leading to high risk of not surviving the first weeks of life. We included a control group of 10 healthy, vaginally delivered full-term control (FTC) infants born at the University Hospital of Northern Norway. Sample size calculation for studies assessing gut microbiota taxonomic composition can be performed by assessing matrices of pairwise distances between groups (23). We expected that around 30 infants in each group of preterm infants would afford 90% statistical power to detect differences in gut microbiota composition that were smaller than effects previously observed in microbiota studies of antibiotic exposure (23). The sample size was also adapted to cover the high expenses for shotgun-metagenome sequencing. The original protocol (24) focused on taxonomic composition. We decided post hoc to add a resistome analysis.

After careful instructions, fecal samples were collected by a nurse in the NICU at around seven and 28 days of age, and by the parents at home at around 4 months of age. We used a commercially available sampling kit (OMNIgen GUT kit, DNA Genotek, Ottawa, Canada) allowing storage of samples at ambient temperatures for up to 14 days before DNA extraction (25). We obtained routine clinical data including details on antibiotic exposure. NEC was defined as Bell's stage 2–3 (26).

### DNA extraction, library preparation, and sequencing

Total metagenomic DNA was extracted using the NorDiag Arrow Stool DNA Extraction kit (NorDiag, Oslo, Norway). An extra beadbeating step was added to facilitate cell lysis as studies have shown that this can increase extraction of DNA from Gram-positive bacteria. DNA was quantified using the Nanodrop 1000 and Qubit^Ⓡ^ 2.0 Fluorometer (Invitrogen, Carlsbad, CA, USA) along with the Qubit^Ⓡ^ dsDNA HR assay kit (Thermo Fisher Scientific, Waltham, MA, USA). DNA was then stored at −70°C. The indexed paired-end libraries were prepared for whole genome sequencing using the Nextera XT Kit (Illumina, San Diego, CA, USA), according to the manufacturer's instructions. Fifty nanograms of genomic DNA was tagmented at 55°C for 10 min. The tagmented DNA was amplified with two primers from Nextera DNA sample preparation Index Kit. PCR products were cleaned using Agencourt AMPure XP beads (Beckman Coulter, Indiana, USA). Purified PCR products were quantified using the Qubit^Ⓡ^ 2.0 (Invitrogen, Carlsbad, CA, USA), along with the Qubit^Ⓡ^ dsDNA HS assay kit (Thermo Fisher Scientific, Waltham, MA, USA). The fragment size distribution (500–1,000 bp) was analyzed using the Agilent 2100 Bioanalyzer System (Agilent Technologies, Waldbronn, Germany). The samples were pooled at concentration of 4 nM per sample. Eight to twelve samples were pooled per each sequencing run. Pooled samples was denatured with 0.2 N NaOH, then diluted to 10 pM with hybridization buffer. Subsequently, samples were submitted for v3 reagents with 2 × 300 cycles paired-end sequencing using the Illumina Miseq platform, according to the manufacturer's instructions. In total, 184 samples were sequenced to an average (range) sequence depth of 4.8 (1.8–12.6) million reads per sample for microbiota and functional analysis. Prior to all downstream data analysis, the sequence quality was calculated using FastQC (v0.11.3). All samples were screened for human contamination using Deconseq with default parameters and build up 38 of the human genome as reference. Quality filtering of the read was performed using Trimmomatic v0.36 with LEADING:3, TRAILING:3, MINLEN:75 as parameter settings. Assemblies were performed on the trimmed reads using MEGAHIT. Functional annotation was added using an in-house genome annotation pipeline, the META-pipe (Department of Chemistry, University of Tromsø, Norway [https://arxiv.org/abs/1604.04103]). The sequences are deposited in the European Nucleotide Archive (www.ebi.ac.uk/ena); study accession nr. PRJEB29052.

### Taxonomic profiling

The relative abundance of bacteria at genus level was calculated from the trimmed reads using MetaPhlAn 2.0 (27). Relative abundance tables for each individual sample were merged. To calculate longitudinal changes, sequences were reconstructed using the Lowest Common Ancestor (LCA) classifier.

### The gut antibiotic resistome

The prediction of genes presumed to confer antibiotic resistance was performed on the assembled metagenomes using Abricate [https://github.com/tseemann/abricate] against the resistance gene identifier in the Comprehensive Antibiotic Resistance Database (CARD; version 1.1.1; Dept. of Biochemistry and Biomedical Science, McMaster University, Canada, https://card.mcmaster.ca/home]) (25–28) with the minimum identity threshold set to 75% (28). Because of the fragmented nature of the metagenome assemblies, and therefore presence of fragmented genes, multiple hits against the same antibiotic resistance gene (ARG) were regarded as one hit. Data are presented as distribution of ARG classes among the three different groups of infants at three time points. Classes of antibiotic resistance genes in the CARD database and the specific genes included in each class are listed below

- Beta lactamase: *blaMIR, blaZ, blaACT, blaTEM, blaCMY, blaLEN, blaADC, blaACI, blaOXA, blaOXY, blaSHV, blaDHA, blaOKP, blaACC, blaSED, blaMOR, blaCMG, blaCFE, cfiA, cepA, cfxA*
- Methicillin resistance: *mecA*
- Aminoglycosides: *aac(6*′*)-aph(2), aac(6*′*)-Ic, aac(6*′*)-Im, aadA, aadB, aadD, aadE, ant(6)-Ia, aph(2)-Ib, aph(3)-Ia, aph(3)-III, spc, str, strA,strB*
- Tetracyclines: *tet(A), tet(B), tet(M), tet(K), tet(X), tet(O), tet(L), tet(U), tet(Q), tet(W), tet(S), tet(32), tet(34), tet(35), tet(37), tet(40), tet(41), Otr(A)*
- Fluoroquinolones: *QnrB, QnrD*
- MLS: Macrolide: *erm(A), erm(B), erm(C), erm(F),erm(G), erm(T), erm(X), mph(A), mph(C); Lincosamide: lnu(B), lnu(C); Streptogranin: vat(B), vat(F)*
- ABC efflux: *lsa(A),lsa(B), lsa(C), msr(A), mrs(C), msr(D), ole(B), car(A)*
- RND efflux pumps: *oqxA*
- Efflux pumps: *vga(A), mef(A)*
- Multidrug efflux pumps: *norA*
- Chloramphenicol: *cat, catA, catB, catS, cmlA, cml*
- Fosfomycin*: fos(A)*
- Sulfonamides: *sul1, sul2*
- Antibiotic target: *dfrA, dfrG*
- Vancomycin: *VanC, VanS, VanT, VanR, VanY*
- Metronidazole: *nimB*

In order to obtain quantitative measures of the putative ARGs in each sample, the quality trimmed reads were analyzed using Short, Better Representative Extract Dataset (ShortBRED) (29) against a formatted CARD database and normalized per total reads in each sample. Data are presented as abundance of ARGs among the three different groups of infants at three time points. Using ShortBRED we identified the antibiotic resistance gene classes and genes listed below:

- Class A Beta lactamase
- Class C Beta lactamase
- Aminoglycoside acetyltransferase
- Aminoglycoside phosphotransferase
- Aminoglycoside nucleotidyltransferase
- Tetracycline efflux
- Tetracycline ribosomal protection
- Quinolone resistance
- Macrolide/MLS resistance
- Adenosine triphosphate (ATP)-binding cassette (ABC) efflux pump
- Resistance/nodulation/division (RND) antibiotic efflux
- Major facilitator superfamily (MFS) antibiotic efflux
- Multidrug efflux pump activity
- Multidrug resistance efflux pump
- Genes modulating antibiotic efflux: *norA, baeR, marA, phoQ, ramA, soxR*
- Small multidrug resistance (SMR) antibiotic efflux
- Chloramphenicol acetyltransferase
- Antibiotic target
- Genes modulating resistance: *WblE, WhiB*
- rRNA methyltransferase
- Other ARG: *bacA*

### Probiotic supplementation

A consensus-based protocol for probiotic supplementation was implemented in Norway in 2014 (30). Extremely preterm infants, contributing to around 90% of NEC cases in Norway, were considered as the target group for probiotic prophylaxis. At this time, probiotics was not used routinely for more mature preterm infants (≥28 weeks gestation) in any Norwegian neonatal unit. After considering the safety profile, a widely used probiotic combination product was selected (Infloran^Ⓡ^) (31). One capsule Infloran contained 10^9^ colony forming units (CFU) *Lactobacillus acidophilus* (ATCC 4356) and 10^9^ CFU *B. longum* subspecies *infantis* (ATCC 15697). One-half capsule once daily was initiated on day 3–4 and increased to one capsule daily after 4–7 days. One capsule was opened and the content was diluted in 2 ml of breast milk, or formula. It was thereafter administered enteral via a nasogastric tube, either 1 ml (1/2 capsule) or 2 ml (one capsule).

### Influence of antibiotic therapy

To quantify changes in the gut microbiota composition and resistome after antibiotic exposure, we stratified four different categories of antibiotic exposure: (i) antenatal exposure, (ii) short (≤72 h) vs. prolonged (>72 h) exposure in the first week of life (19, 22), (iii) any exposure after first week of life (yes/no), and (iv) narrow- vs. broad-spectrum exposure after first week of life. Potential effects of antenatal exposure and short vs. prolonged therapy after birth were only investigated at 7 days of age. We defined regimens including third-generation cephalosporins or carbapenems as a broad-spectrum regimens when compared to regimens containing aminoglycosides for coverage against Gram-negative bacteria. This definition was based on the fact that neonatal empiric treatment using a third-generation cephalosporin for Gram-negative coverage induce significantly higher antibiotic resistance rates among colonizing bacteria than a regimen containing an aminoglycoside (32).

### Ethics, trial registration, and statistical analysis

The study was approved by the Norwegian Regional Ethical Committee (2014/930/REK nord) and registered in Clinicaltrials.gov (https://clinicaltrials.gov/ct2/show/NCT02197468). Informed written consent was obtained from all parents.

Data were analyzed using IBM-SPSS version 22 (IBM, Armonk NY, USA) statistical software, the R statistical framework (version 3.2.4; http://www.r-project.org/), and Statistical Analysis of Metagenomic Profiles (STAMP) software package (33). We used Mann–Whitney *U*-test or a Kruskal–Wallis test for comparisons between two or multiple independent groups. We used a Poisson generalized linear model to calculate trends in the relative abundance of genera and ARGs in the gut microbiota. Corrections based on multiple comparisons were performed by the Benjamini–Hochberg false discovery rate (FDR) (34). A FDR *Q* ≤ 0.10 was considered significant for any analyses with multiple comparisons. A standard *P* ≤ 0.05 was considered significant for all other analyses.

Alpha diversity was assessed by calculating the Shannon Diversity index (MEGAN, v5.10.6) (35). To detect changes in alpha diversity over time, we first performed a normality test and found that the residuals were normally distributed. Therefore, differences in alpha diversity over time between the three different groups were calculated using linear mixed models. The same model was used to calculate the influence of antibiotic exposure on alpha diversity. Multiple beta diversity metrics of samples was performed using non-metrical multidimensional scaling (NMDS) based on a matrix of Bray-Curtis distances calculated using the vegan R package. Differences between groups were tested using permutational multivariate analysis on beta diversity matrices.

## Results

### Study population and antibiotic exposure

**Figure 1:**
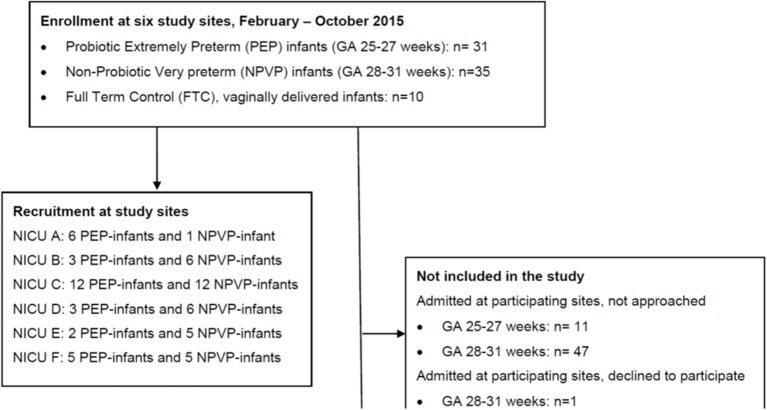
CONSORT study flow diagram. PEP, probiotic extremely preterm; NPVP, non-probiotic very preterm; FTC, full term control; NICU, Neonatal Intensive care Unit.

Figure 1 shows study flow. We enrolled 66 preterm infants and 10 healthy full-term control (FTC) infants between February and October 2015. The six study sites had different admission numbers, and recruited each between 7 and 24 preterm infants (Figure 1). Clinical characteristics, antibiotic and probiotic exposure, duration of parenteral nutrition and enteral nutrition data are reported in Table 1. The “probiotic extremely preterm (PEP)” infants received much more antibiotics than the “non-probiotic very preterm (NPVP)” infants after first week of life. Two infants in the PEP-group were operated for NEC, both survived.

**Table 1:** Clinical background data.

	**Probiotic extremely preterm (PEP) infants**	**Non-probiotic very preterm (NPVP) infants**	**Full term control (FTC) infants**
	**(*n* = 31)**	**(*n* = 35)**	**(*n* = 10)**
Birth weight [grams], median (IQR)	835 (680–945)	1,290 (1,150–1,445)	3,613 (3,394–3,733)
Gestational age [weeks], median (IQR)	26 (26–27)	30 (29–30)	40 (40–41)
Gender			
Male, *n* (%)	13 (42%)	20 (57%)	3 (30)
Female, *n* (%)	18 (58%)	15 (43%)	7 (70)
Route of delivery			
Cesarean, *n* (%)	21 (68%)	20 (57%)	0 (0)
Vaginal, *n* (%)	10 (32%)	15 (43%)	10 (100)
CRIB score, mean (SD)	11 (2)	5 (2)	–
Any antenatal antibiotic exposure, *n* (%)	8 (26%)	12 (34%)	0 (0)
Any antibiotic exposure first week of life*, *n* (%)	30 (97%)	27 (77%)	–
Median (IQR) days—antibiotics exposed infants	6 (4–7)	4 (3–5)	–
Any antibiotic exposure after first week of life, *n* (%)	22 (71%)	5 (14%)	–
Narrow spectrum regimen after first week of life, *n* (%)	14 (45%)	3 (9%)	–
Broad-spectrum** regimen after first week of life, *n* (%)	8 (26%)	2 (5%)	–
Median (IQR) days antibiotics in exposed infants	6.5 (3–13)	10 (5.5–14)
Total days antibiotics, median (IQR); antibiotics exposed infants, *n*	9.5 (6–18) *n* = 30	4 (3–6) *n* = 27	–
Total days of probiotic supplementation, median (IQR)	46 (40–57)	–	–
Parenteral nutrition, *n* (%)	31 (100%)	16 (46%)	–
Median (IQR) days parenteral nutrition	9 (6–13)	5 (3–8)	–
Exclusive human milk nutrition until discharge	17 (55%)	16 (46%)	10 (100)

*CRIB, Clinical Risk Index for Babies; IQR, interquartile range*.

* *Only ampicillin or penicillin + gentamicin were used in all preterm infants in first week of life*.

** *We defined regimens including third-generation cephalosporins or carbapenems as a broad-spectrum regimen*.

### Taxonomic composition

**Figure 2:**
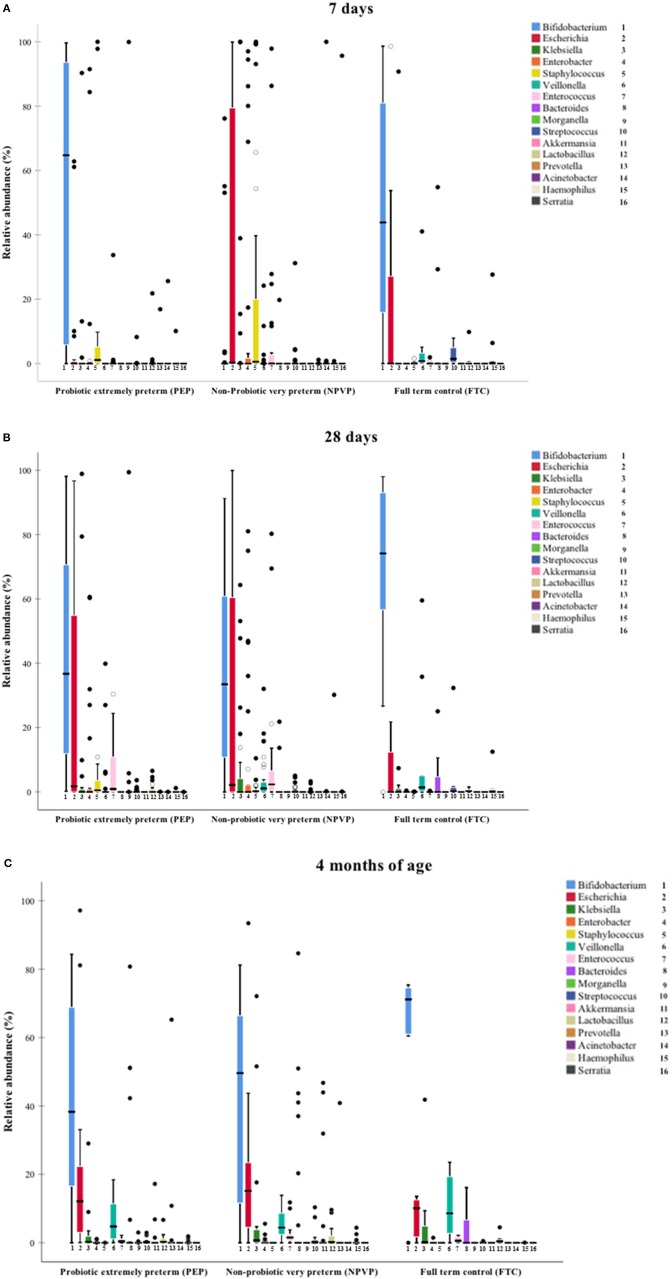
**(A–C)** Median relative abundance of dominant taxa at genus level. Box plot diagram where the inside bar represent median, the outer horizontal line of the box represents the 25th and the 75th percentile. **(A)** Median relative abundance at 7 days. **(B)** Median relative abundance at 28 days. **(C)** Median relative abundance at 4 months.

On day 7, we found higher relative abundance of *Bifidobacterium* and *Lactobacillus* in PEP-infants compared to NPVP-infants (Figure 2A, Table 2). FTC infants had higher abundance of some genera (*Streptococcus, Veilonella*, and *Haemophilus*) that were only sparsely present in the two preterm infant groups (Figure 2A). Mode of delivery did not lead to detectable differences in the microbiota composition within the preterm groups on day 7 (data not shown).

**Table 2:** Median relative abundance (%) of dominant genera in infant gut microbiota at 7, 28 days, and 4 months of age.

	**7 days (*****n*** = **60 fecal samples)**					**28 days (*****n*** = **64 fecal samples)**					**4 months (*****n*** = **60 fecal samples)**				
**Genus**	**PEP**	**NPVP**	**FTC**	***P*-value**	**FDR Q**	**PEP**	**NPVP**	**FTC**	***P*-value**	**FDR**	**PEP**	**NPVP**	**FTC**	***P*-value**	**FDR Q**
	**(*n* = 20)**	**(*n* = 30)**	**(*n* = 10)**			**(*n* = 24)**	**(*n* = 31)**	**(*n* = 9)**		**Q**	**(*n* = 24)**	**(*n* = 29)**	**(*n* = 7)**		
*Bifidobacterium*	64.7	0.00^***^	43.9	<**0.001**	<**0.001**	36.7	33.5	74.1	0.088	0.156	38.3	49.6	71.2	0.243	0.555
*Escherichia*	0.00	0.27	0.02	0.107	0.245	1.76	2.10	0.00	0.351	0.511	12.1	15.2	10.10	0.377	0.754
*Klebsiella*	0.00	0.00	0.00	0.737	0.786	0.00	0.00	0.00	0.663	0.816	0.25	0.67	0.11	0.738	1.0
*Enterobacter*	0.00	0.00	0.00	0.125	0.222	0.00	0.00	0.00	0.225	0.360	0.00	0.00	0.00	0.110	0.440
*Staphylococcus†*	1.10	0.54	0.05	0.230	0.368	0.51	0.23	0.01^*^	**0.038**	**0.076**	0.00	0.00	0.00	0.472	0.839
*Veilonella†*	0.00	0.00^*^	0.75^***^	<**0.001**	<**0.001**	0.00	1.09^*^	1.38^*^	**0.018**	**0.072**	4.75	4.44	8.59	0.812	1.0
*Enterococcus†*	0.00	0.01	0.00	0.118	0.236	0.90	2.35	0.00^*^	**0.003**	**0.016**	0.39	1.53^**^	0.58	0.019	0.152
*Bacteroides†*	0.00	0.00	0.00	**0.005**	**0.013**	0.00	0.00	0.00	**0.001**	**0.008**	0.00	0.00	0.00	0.996	1.0
*Morganella*	0.00	0.00	0.00	0.368	0.535	0.00	0.00^*^	0.00	**0.030**	**0.069**	0.00	0.00	0.00	0.098	0.523
*Streptococcus*	0.00	0.00	1.45^***^	<**0.001**	<**0.001**	0.00	0.06^*^	0.26^*^	**0.018**	**0.058**	0.15	0.14	0.06	0.149	0.477
*Akkermansia*	0.00	0.00	0.00	1.0	1.0	0.00	0.00	0.00	1.00	1.0	0.00	0.00	0.00	0.171	0.456
*Lactobacillus*	0.00	0.00^*^	0.23	**0.004**	**0.013**	0.00	0.00	0.23	**0.019**	**0.051**	0.26	0.18	0.42	0.682	1.0
*Prevotella†*	0.00	0.00	0.00	0.716	0.818	0.00	0.00	0.00	0.435	0.580	0.00	0.00^**^	0.00	**0.001**	**0.016**
*Acinetobacter*	0.00	0.00	0.00	0.525	0.70	0.00	0.00	0.00	0.834	0.953	0.00	0.00	0.00	1.000	1.0
*Haemophilus*	0.00	0.00	0.14^*^	<**0.001**	<**0.001**	0.00	0.00	0.07^**^	<**0.001**	<**0.001**	0.00	0.00	0.00	0.996	1.0
*Serratia*	0.00	0.00	0.00	0.607	0.747	0.00	0.00	0.00	0.834	0.890	0.00	0.00	0.00	1.000	1.0

*PEP, probiotic extremely preterm; NPVP, non-probiotic very preterm; FTC, full term control; FDR, false discovery rate*.

*Dominant genera have an overall median relative abundance > 0.5% at 7 days, 28 days, and 4 months of age*.

Overall comparison of all three treatment groups at each time point by non-parametric Kruskal–Wallis test. Post-hoc comparisons by non-parametric Mann–Whitney U–test (NPVP or FTC vs. PEP) (^*x**^P < 0.001, ^**^P < 0.01, ^*^P < 0.05).

† *Comparison between the three different time points was by a generalized linear model with a Poisson family (P < 0.05)*.

*Bold indicates significant differences in median relative abundance of bacterial genera between the three groups (P- and Q-value)*.

On day 28, there was a striking increase in relative abundance of *Escherichia* in the PEP-infants and a similar striking increase in relative abundance of *Bifidobacterium* in NPVP-infants. FTC infants had significantly higher relative abundance of *Lactobacillus* than NPVP-infants. Overall, at 28 days of age the FTC- and NPVP-infants had higher abundance of *Veilonella* and *Streptococcus* than PEP-infants, while both preterm groups had higher relative abundance of *Staphylococcus* and *Enterococcus* than FTC-infants (Figure 2B).

By 4 months of age, there were no significant differences in taxonomic profile between PEP- and FTC-infants. The NPVP-infants had more *Prevotella* than PEP-infants, but otherwise all three groups were similar (Figure 2C). Duration of parenteral nutrition did not lead to detectable differences in the microbial composition between the preterm group(s) on 28 days and at 4 months of age (data not shown). We found no differences in abundance of bifidobacteria and or lactobacilli between hospitals at any time point.

### Influence of antibiotic exposure on taxonomic composition

We found no significant influence of antenatal antibiotic exposure on the gut microbiota composition on day 7. However, 57/66 (86%) preterm infants also received antibiotic therapy (ampicillin or penicillin + gentamicin) during the first week of life (Table 1), limiting the possibility to detect isolated effects of antenatal exposure. There was no difference in the gut microbiota between those exposed to a short (≤72 h) compared to a prolonged (>72 h) course during first week of life. Broad-spectrum antibiotic therapy after the first week of life was mainly given to PEP-infants. Only one child in the NPVP-group received third generation cephalosporins after first week of life. At 4 months of age there was reduced relative abundance of *Lactobacillus* and *Veilonella* in those exposed to broad-spectrum antibiotics compared to infants exposed to narrow-spectrum therapy (Tables 3, 4). Moreover, there was a non-significant trend toward reduced relative abundance of *Bifidobacterium* and increased relative abundance of *Escherichia* among all preterm infants exposed to broad-spectrum antibiotics at both 28 days and 4 months of age (Tables 3, 4).

**Table 3:** Influence of antibiotic exposure (broad^*^ vs. narrow) on taxonomic composition in all preterm infants (both PEP- and NPVP-infants) with fecal samples and who received antibiotics after first week of life.

	**Microbiota at 28 days**			**Microbiota at 4 months**			
	**Median relative abundance**			**Median relative abundance**			
**Antibiotic regimen**	Broad*	**Narrow**	***P***	Broad*	**Narrow**	***P***	**FDR Q**
	(*n* = 7**)	(*n* = 15**)		(*n* = 9**)	(*n* = 13**)		
**BACTERIAL GENERA**							
*Bifidobacterium*	14.4	28.9	0.783	14.3	41.5	0.096	0.512
*Escherichia*	44.5	1.40	0.368	17.4	9.9	0.209	0.669
*Klebsiella*	0.00	0.00	0.680	0.25	0.57	0.845	0.623
*Enterobacter*	0.00	0.45	0.123	0.00	0.00	0.235	0.627
*Staphylococcus*	0.42	0.08	0.783	0.00	0.00	1.00	1.00
*Veilonella*	0.00	0.00	0.945	1.25	6.01	**0.001**	**0.016**
*Enterococcus*	2.73	0.68	0.783	0.64	0.39	0.647	1.00
*Streptococcus*	0.00	0.00	0.630	0.07	0.18	0.126	0.504
*Lactobacillus*	0.00	0.00	0.891	0.00	0.87	0.071	0.568

*PEP, probiotic extremely preterm; NPVP, non-probiotic very preterm*.

* *We defined regimens including third-generation cephalosporins or carbapenems as a broad-spectrum regimen*.

** *Number of fecal samples included in these analyses*.

*Median relative abundance of Bacteroides, Morganella, Akkermansia, Prevotella, Acinetobacter, Haemophilus, and Serratia were < 0.001 at 28 days and 4 months of age and there were no statistical difference between groups*.

*Bold indicate significant difference between broad- and narrow-spectrum antibiotic exposure*.

*FDR, false discovery rate; only calculated for comparisons with P < 0.05*.

**Table 4:** Influence of antibiotic exposure (broad^*^ vs. narrow) on taxonomic composition in only the PEP-infants with fecal samples and who received antibiotics after first week of life.

	**Microbiota at 28 days**			**Microbiota at 4 months**			
	**Median relative abundance**			**Median relative abundance**			
**Antibiotic regimen**	Broad*	**Narrow**	***P***	Broad*	**Narrow**	***P***	**FDR Q**
	(*n* = 5**)	(*n* = 12**)		(*n* = 7**)	(*n* = 11**)		
**BACTERIAL GENERA**							
*Bifidobacterium*	14.39	32.50	0.574	14.31	45.96	0.035	0.187
*Escherichia*	44.54	0.69	0.160	33.06	9.88	0.179	0.477
*Klebsiella*	0.00	0.00	0.721	0.26	0.57	1.000	1.00
*Enterobacter*	0.00	0.52	0.195	0.00	0.00	0.143	0.572
*Staphylococcus*	0.42	0.36	0.879	0.00	0.00	1.000	1.000
*Veilonella*	0.00	0.00	0.506	0.96	6.01	**0.004**	**0.064**
*Enterococcus*	2.73	0.15	0.506	0.33	0.40	0.536	0.858
*Streptococcus*	0.54	0.00	0.442	0.07	0.14	0.285	0.651
*Lactobacillus*	0.00	0.00	0.959	0.00	1.21	**0.004**	**0.032**

*PEP, probiotic extremely preterm*.

* *We defined regimens including third-generation cephalosporins or carbapenems as a broad-spectrum regimen*.

** *Number of fecal samples included in these analyses*.

*Median relative abundance of Bacteroides, Morganella, Akkermansia, Prevotella, Acinetobacter, Haemophilus, and Serratia were <0.001 at 28 days and 4 months of age and there were no statistical difference between groups*.

*Bold indicate significant difference between broad- and narrow-spectrum antibiotic exposure*.

*FDR, false discovery rate; only calculated for comparisons with P < 0.05*.

### Diversity of the gut microbiota and influence of antibiotic exposure

**Figure 3:**
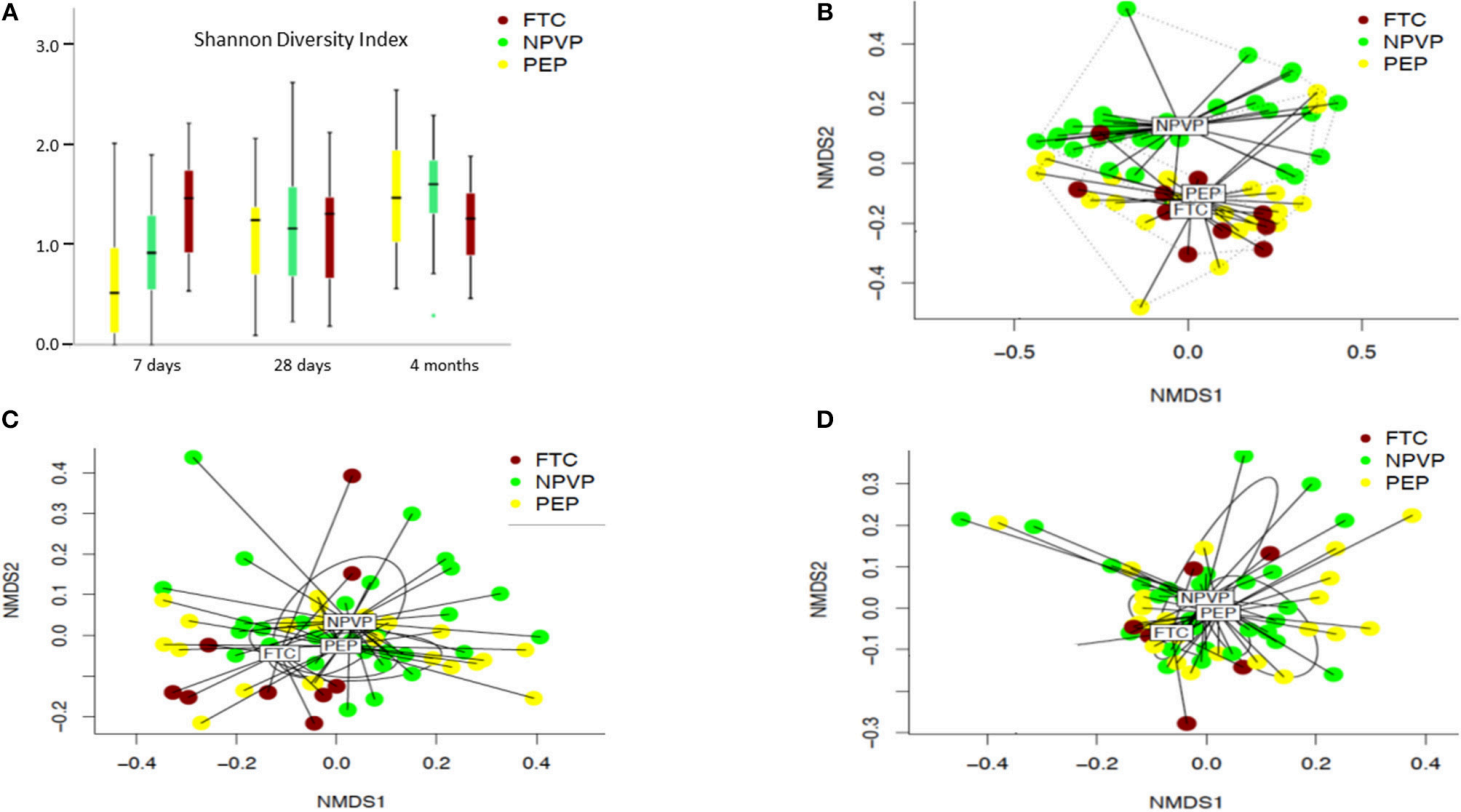
**(A–D)** Alpha diversity calculated by Shannon diversity index and beta diversity between PEP, NPVP, and FTC infants calculated by non-metrical multidimensional scaling (NMDS). Box plot diagram where the inside bar represent median, the outer horizontal line of the box represents the 25th and the 75th percentile. Error bars represent the standard error. Differences between groups at a given time point and at different time points were tested with linear mixed model. **(A)** Shannon diversity index of three groups of infants at three sampling points. **(B)** Beta diversity (NMDS) at 7 days. **(C)** Beta diversity (NMDS) at 28 days. **(D)** Beta diversity (NMDS) at 4 months.

We found large intra-individual differences in the gut microbiota composition, in particular at 7 and 28 days of age (Figures 2A–C). The alpha diversity increased significantly with age in both preterm infant groups, but not in FTC-infants (Figure 3A). FTC-infants had significant higher diversity compared to PEP infants at 7 days of age. On day 28 and at 4 months of age, there were no significant differences in alpha diversity between any groups. Significant overall community (beta diversity) differences using Bray-Curtis dissimilarity were detected comparing the three groups on infants (PEP, NPVP, and FTC) at 7 days of age (*P* = 0.001) and 28 days of age (*P* = 0.003) (Figures 3B–D). However, we found no difference in alpha or beta diversity between different categories of antibiotic exposure at the three sampling time points.

### Antibiotic resistome–distribution of ARG classes and abundance of ARGs

In all three groups, we identified putative ARGs conferring resistance to nine different classes of antibiotics, including beta lactams, aminoglycosides, tetracyclines, fosfomycine, sulphonamides, vancomycin, and the macrolide-lincosamide-streptogramin B group. Genes conferring resistance to fluoroquinolones and chloramphenicol were only detected in PEP- and NPVP-infants. Several genes encoding efflux pumps were also identified at all three sampling time points. In total 99 unique ARGs were identified, of which 28 (28%) were located on mobile genetic elements, and these latter were found in more than 80% of all infants (Table 5).

**Table 5:** Distribution of classes of antibiotic resistance genes among infants in each group.

Antibiotic group or resistance mechanism**	**7 days**			**28 days**			**4 months**		
	**PEP**	**NPVP**	**FTC**	**PEP**	**NPVP**	**FTC**	**PEP**	**NPVP**	**FTC**
	*n* = 20*	*n* = 30*	*n* = 10*	*n* = 24*	*n* = 31*	*n* = 9*	*n* = 24*	*n* = 29*	*n* = 7*
Beta lactamases	10/20	24/30	3/10	19/24	22/31	6/9	18/24	25/29	4/7
MecA gene	9/20	11/30	–	5/24	5/31	–	–	–	–
Aminoglycoside	8/20	14/30	3/10	11/24	16/31	2/9	12/24	16/29	2/7
Tetracycline	9/20	22/30	8/10	17/24	30/31	9/9	23/24	29/29	7/7
Fluoroquinolones	–	1/30	–	1/24	–	–	3/24	4/29	–
Macrolides	7/20	5/30	2/10	6/24	2/31	–	2/24	–	–
MLS	3/20	9/30	3/10	4/24	11/31	3/9	8/24	15/29	4/7
ABC efflux pumps	6/20	7/30	–	16/24	24/31	4/9	17/24	23/29	7/7
RND efflux pumps	7/20	12/30	2/10	12/24	18/24	4/9	12/24	19/24	5/7
Efflux pumps	3/20	3/30	8/10	2/24	4/31	2/9	6/24	8/24	3/7
Multidrug Efflux pump	9/20	14/30	1/10	11/24	7/31	1/9	–	–	–
Chloramphenicol	3/30	9/30	–	6/24	7/31	–	9/24	3/29	–
Fosfomycine	18/20	21/30	3/10	22/24	25/31	5/9	20/24	27/29	4/7
Sulfonamides	2/20	3/30	–	6/24	7/31	–	10/24	9/29	2/7
Antibiotic target	1/20	1/30	–	4/24	4/31	–	6/24	3/29	3/7
Antibiotic inactivation	–	2/30	1/10	1/24	1/31	–	6/24	7/29	2/7
Vancomycin	–	–	–	–	–	–	5/24	8/29	3/7
Metronidazole	–	–	–	–	–	–	–	1/29	–

*PEP, probiotic extremely preterm; NPVP, non-probiotic very preterm; FTC, full term control*.

* *Number of fecal samples included in these analyses*.

** *See Methods for further explanation of which antibiotic resistance genes that are included in these groups*.

We found 21 different genes encoding beta-lactamases, including broad-spectrum and extended-spectrum beta lactamases (ESBLs). ESBL-genes were represented at all three time points in NPVP- and FTC-infants, but not detected in PEP-infants. The methicillin resistance gene (*mecA*) was identified at 7 and 28 days of age in 11/35 NPVP-infants and 13/31 PEP-infants, but not at 4 months of age. Only one PEP-infant and four NPVP-infants were persistent fecal carriers of *mecA* at days 7 and 28. Vancomycin ARGs were identified at 4 months of age in 16 infants, but only four of these had received vancomycin. Many of the ARGs identified, encoded resistance to other antibiotics than those used in the NICUs.

On day 7 NPVP-infants had higher abundance of ARGs from four different ARG classes and PEP-infants higher abundance of ARGs from two other ARG classes (Table 6). Only 24% of ARG-classes changed significantly their abundance during the three sampling points (*P* < 0.05) (Table 6).

**Table 6:** Median abundance of antibiotic resistance genes among infants in each group.

**Antibiotic resistance genes (ARG) encoding Classes of ARG**	**7 days (*****n*** = **60 fecal samples)**					**28 days (*****n*** = **64 fecal samples)**					**4 months (*****n*** = **60 fecal samples)**				
	**PEP**	**NPVP**	**FTC**	***P***	**FDR Q**	**PEP**	**NPVP**	**FTC**	***P***	**FDR Q**	**PEP**	**NPVP**	**FTC**	***P***	***FDR* Q**
	**(*n* = 20)**	**(*n* = 30)**	**(*n* = 10)**			**(*n* = 24)**	**(*n* = 31)**	**(*n* = 9)**			**(*n* = 24)**	**(*n* = 29)**	**(*n* = 7)**	
Class A Beta lactamase	0.61	4.2^*^	0.00^*^	**0.001**	**0.020**	0.00	0.00	0.00	0.080	0.586	1.43	1.0	0.00	0.443	1.327
Class C Beta lactamase	0.00	0.00	0.20	0.126	0.229	0.98	0.22	0.00	0.492	0.812	9.1	12.7	9.5	0.605	1.134
Aminoglycoside acetyltransferase	0.00	0.00	0.00	0.202	0.311	–	–	–	–	–	–	–	–	–	–
Aminoglycoside phosphotransferase	0.00	0.00	0.00	0.590	0.653	0.00	0.16	0.00	0.114	0.497	–	–	–	–	–
Aminoglycoside nucleotidyltransferase	0.00	0.00	0.00	0.765	0.765	0.00	0.00	0.00	0.296	0.426	0.00	0.00	0.00	0.584	0.814
Tetracycline efflux	0.00	0.00^*^	0.00	**0.015**	**0.050**	0.00	0.00	0.00	0.173	0.423	0.00	0.00	0.00	0.174	1.949
Tetracycline ribosomal protection	0.00	0.26	4.4^*^	**0.047**	0.118	0.52	3.7	1.77	0.397	0.615	6.4	23.4	23.4	0.407	1.041
Quinolone resistance^†^	9.0	21.6	5.3	0.062	0.138	9.81	7.6	0.77	0.133	0.470	9.2	9.4	7.1	0.501	1.186
Macrolide/MLS resistance	0.00	0.00	0.00	0.757	0.797	–	–	–	–	–	–	–	–	–	–
ABC efflux pump^†^	0.13	1.15	0.25	0.206	0.294	1.06	1.35	0.06^*^	0.013	0.414	0.70	0.96	0.83	0.766	0.887
RND antibiotic efflux	5.2	41.9^*^	38.4	**0.034**	**0.097**	37.7	53.7	4.1	0.170	0.683	94.0	116.7	90.3	0.674	0.936
MFS antibiotic efflux	1.16	113.3	29.0	0.339	0.342	85.8	119.1	16.0	0.056	0.489	105.2	119.5	84.7	0.614	0.839
Multidrug efflux pump activity	0.00	24.6	1.92	0.337	0.449	20.9	21.7	4.9	0.346	0.478	10.0	14.0	8.1	0.616	1.552
Multidrug resistance efflux pump	0.00	0.00	0.00	0.668	0.742	0.00	0.00	0.00	0.603	0.678	0.18	0.00	0.60	0.496	0.819
Gene modulating antibiotic efflux	5.6	41.0^**^	0.76	**0.012**	**0.060**	14.7	20.1	0.34	0.163	0.376	19.7	27.7	27.5	0.645	0.871
SMR antibiotic efflux	–	1.2	–	–	–	0.00	0.00	0.00	0.914	0.932	–	–	–	–	–
Chloramphenicol acetyltransferase	0.00	0.00	0.00	**0.071**	0.142	–	–	–	–	–	–	–	–	–	–
Antibiotic target^†^	0.48	0.00	0.00^**^	**0.013**	**0.052**	0.00	0.00	0.00	0.266	0.396	0.00	0.00	0.00	0.720	0.768
Gene modulating resistance	53.5	8.1^**^	39.2	**0.003**	**0.030**	37.6	27.8	44.6	0.419	0.419	37.5	45.8	46.2	0.678	1.286
rRNA methyltransferase^†^	0.00	10.6	10.6	0.128	0.213	6.0	8.8	1.72	0.008	0.464	4.1	5.4	4.4	0.665	0.887
Other ARG^†^	5.3	16.7^**^	2.02	**0.011**	**0.073**	7.3	8.4	0.26	0.132	0.413	7.2	10.5	6.3	0.613	

*Numbers are presented as median total reads normalized by the total number of reads in each fecal sample*.

*Antibiotic resistance genes analyzed using ShortBRED*.

*PEP, probiotic extremely preterm infants; NPVP, non-probiotic very preterm infants; FTC, full-term control; FDR, false discovery rate*.

*Comparisons between all three treatment groups by nonparametric Kruskal–Wallis test*.

Post-hoc comparisons by non-parametric Mann–Whitney U-test (vs. PEP) (^**^P < 0.01, ^*^P < 0.05).

*Comparison between different time points by generalized linear model with a Poisson family (†P < 0.05)*.

*Genes modulating antibiotic efflux: norA, baeR, marA, phoQ, ramA, soxR. Genes modulating resistance: WblE, WhiB. Other ARG: bacA*.

*Bold indicates significant differences in median abundance of antibiotic resistance genes between the three groups (P- and Q-value)*.

On day 7 and at 4 months of age, different antibiotic exposure did not result in significant difference in total abundance of ARGs. However, on day 28, we detected significantly higher abundances of four classes of ARGs, including genes encoding beta-lactam and aminoglycoside resistance, in preterm infants exposed to broad-spectrum antibiotics compared to infants treated with narrow-spectrum regimens (Table 7). For the subset of preterm infants given probiotics there were no significant differences in abundance of ARGs at 4 weeks and 4 months (Table 8).

**Table 7:** Influence of antibiotic exposure (broad vs. narrow spectrum regimen after first week of life) on abundance of antibiotic resistance genes (ARGs) in all preterm infants.

Antibiotic resistance gene (ARG) classes***	**ARGs at 28 days**				**ARGs at 4 months**			
	**Absolute counts/total abundance**				**Absolute counts/total abundance**			
	Broad*	**Narrow**	***P***	**FDR *Q***	Broad*	**Narrow**	***P***	**FDR *Q***
	(*n* = 7**)	(*n* = 15**)			(*n* = 9**)	(*n* = 13**)	
Class A Beta lactamase	0.00	0.00	0.447	0.731	5.00	3.01	0.324	0.864
Class C Beta lactamase	44.96	0.00	**0.021**	**0.095**	9.11	8.16	0.235	0.752
Aminoglycoside phosphotransferase	6.14	0.00	0.078	0.281	–	–	–	–
Aminoglycoside nucleotidyltransferase	0.93	0.00	**0.008**	**0.072**	0.00	0.00	0.794	0.851
Tetracycline efflux	52.29	0.00	**0.014**	**0.084**	7.92	0.00	0.235	0.94
Tetracycline ribosomal protection	5.97	0.00	0.210	0.540	11.68	2.17	0.393	0.886
Quinolone resistance	29.75	9.43	0.298	0.671	9.40	8.34	0.357	0.816
ABC efflux pump	3.23	1.07	0.392	0.784	0.70	0.64	0.471	0.814
RND antibiotic efflux	312.10	37.73	0.875	0.875	94.00	84.96	0.393	0.63
MFS antibiotic efflux	272.36	117.02	0.490	0.68	119.50	107.51	0.404	0.59
Multidrug efflux pump activity	22.08	26.53	0.581	0.70	19.08	13.63	0.647	0.69
Multidrug resistance efflux pump	0.00	0.00	0.162	0.486	3.02	0.00	0.017	0.272
Gene modulating antibiotic efflux	75.30	15.53	0.490	0.73	19.65	20.86	0.393	0.63
SMR antibiotic efflux	0.00	0.00	0.447	0.805	–	–	–	–
Antibiotic target	1.70	0.00	**0.002**	**0.030**	2.36	0.00	0.096	0.512
Gene modulating resistance	16.25	22.83	0.535	0.69	9.68	39.10	0.043	0.344
rRNA methyltransferase	8.59	9.07	0.581	0.65	8.41	5.56	0.601	0.67
Other ARG	24.40	12.15	0.680	0.72	7.21	7.36	0.601	0.74

* *We defined regimens including third-generation cephalosporins or carbapenems as a broad-spectrum regimen*.

** *Number of fecal samples included in these analyses*.

*** *See Materials and Methods section for further explanation of which antibiotic resistance genes that are included in these groups*.

*FDR, false discovery rate*.

*Bold indicates significant differences in abundance of antibiotic resistance genes between broad- and narrow-spectrum regimens (P- and Q-value)*.

**Table 8:** Influence of antibiotic exposure (broad vs. narrow after first week of life) on abundance of antibiotic resistance genes (ARGs) in probiotic supplemented extremely preterm (PEP) infants.

Antibiotic resistance genes (ARGs) classes***	**ARGs at 28 days**				**ARGs at 4 months**			
	**Absolute counts/total abundance**				**Absolute counts/total abundance**			
	Broad*	**Narrow**	***P***	**FDR Q**	Broad*	**Narrow**	***P***	**FDR Q**
	(*n* = 5**)	(*n* = 12**)			(*n* = 7**)	(*n* = 11**)		
Class A Beta lactamase	0.00	0.00	0.799	0.846	1.43	3.01	0.596	0.867
Class C Beta lactamase	45.96	0.00	0.009	0.162	9.11	9.52	0.328	0.875
Aminoglycoside phosphotransferase	6.14	0.00	0.082	0.369	–	–	–	–
Aminoglycoside nucleotidyltransferase	0.93	0.00	0.104	0.312	0.00	0.00	0.860	
Tetracycline efflux	29.55	0.00	0.019	0.171	7.92	7.92	0.375	0.857
Tetracycline ribosomal protection	6.49	0.00	0.082	0.369	11.68	28.48	0.246	0.787
Quinolone resistance	29.75	7.08	0.506	0.828	9.40	9.40	0.425	0.85
ABC efflux pump	3.23	0.43	0.279	0.628	0.70	1.10	0.479	0.852
RND antibiotic efflux	312.10	19.81	0.799	0.900	94.00	93.09	0.536	0.858
MFS antibiotic efflux	272.36	79.67	0.506	0.759	70.92	111.28	0.860	0.917
Multidrug efflux pump activity	22.08	24.71	0.879	0.879	19.08	6.55	0.647	0.863
Multidrug resistance efflux pump	0.00	0.00	0.234	0.602	3.02	3.02	0.069	0.368
Gene modulating antibiotic efflux	75.30	13.81	0.328	0.656	19.65	24.88	0.008	0.128
SMR antibiotic efflux	0.00	0.00	0.506	0.759	–	–	–	–
Antibiotic target	1.70	0.00	0.064	0.030	2.36	0.00	0.151	0.604
Gene modulating resistance	16.25	33.15	0.442	0.756	9.68	60.81	0.043	0.344
rRNA methyltransferase	5.15	6.23	0.799	0.846	8.41	2.85	0.930	0.930
Other ARG	24.40	7.31	0.506	0.700	7.21	7.21	0.724	0.891

* *We defined regimens including third-generation cephalosporins or carbapenems as a broad-spectrum regimen*.

** *Number of fecal samples included in these analyses*.

*** *See Materials and Methods section for further explanation of which antibiotic resistance genes that are included in these groups*.

*FDR, false discovery rate. Aminoglycoside acetyltransferase, Macrolide resistance genes, Chloramphenicol acetyltransferase were only present at 7 days of age*.

## Discussion

The main aim of this explorative, observational multi-center study was to obtain in-depth knowledge on how probiotics and antibiotic therapy influenced the developing gut microbiota and antibiotic resistome of preterm infants. Previous studies have shown that the gut microbiota in preterm infants differs from term infants with limited diversity and delayed acquisition of a stable profile (36–38). However, most studies have assessed the gut microbiota composition collapsed at higher taxonomic rank levels (above species-genera level) by sequencing of the 16S ribosomal RNA gene (31, 39). There is limited data (21) on the association between use of probiotics, antibiotics and gut resistome development using shotgun-metagenomic sequencing.

Bifidobacteria strongly dominated the gut microbiota in extremely preterm infants only few days after commencing probiotic supplementation, in sharp contrast to very preterm infants not receiving probiotics who predominantly had *Escherichia*. High levels of probiotic bacteria are not necessarily indicative of colonization, but may represent the passage of DNA from the administered probiotic species through the host (40). Still, early dominance of bifidobacteria may theoretically enhance the risk of translocation to the blood stream, in particular during first weeks of life in extremely preterm infants when enteral nutrition with “fuel for bifidobacteria” is not yet fully established (13, 14). However, bifidobacterial infections are usually mild (14, 41), in contrast to sepsis caused by Gram-negative bacteria (*Proteobacteria*), which in preterm infants are the first colonizers of the intestinal tract. Previous studies have shown that the gut microbiota of preterm infants shortly after birth have a high proportion of *Proteobacteria* and that a bloom of *Bifidobacterium* occurs first around 33 weeks of age, in line with our findings in NPVP-infants at 7 and 28 days of age (42, 43).

*Lactobacillus* was only detected in small amounts in all groups, but relative abundance increased up to 4 months of age in all three groups. High levels of *Bifidobacterium* and barely detectable levels of *Lactobacillus* have been reported earlier in infants supplemented with equal doses of a probiotic combination of bifidobacteria and lactobacilli (31). A possible explanation for this observation is the spatial organization of intestinal bacteria, where lactobacilli are found in intestinal crypts, thus less accessible when collecting luminal contents (44). Indeed, a recent study in adults showed marked differences between the small intestine microbiota compared to the colonic microbiota (45), indicating the scientific limitations with fecal samples when aiming to understand the entire human intestinal ecosystem.

There is no consensus on the optimal dose of probiotics. One study from India compared standard and high-dose probiotic regimens and found no difference in proportion of infants colonized or quantitative colonization rates with probiotic species (46). Most large randomized trial have used daily doses of 1 × 10^8^-10^9^ CFU (40, 47, 48). Some authors suggest that at least 1 × 10^9^ CFU is required to achieve a beneficial effect, in line with the doses used in our study (49). We observed an early and high relative abundance of *Bifidobacterium* in PEP-infants. However, we did not use traditional microbiological methods to assess the overall bacterial abundance in the gut. Some authors have suggested that a gradual increase in probiotic supplementation concomitantly with increased enteral nutrition may replicate the physiological gut microbiota development, and secure gut growth, digestive maturation and an appropriate response to bacterial colonization (50, 51). Our study does not allow us to draw any conclusions on dosing. A recent study reported that a daily dose of the same probiotic used in our study (Infloran^Ⓡ^) leads to significantly higher levels of *Bifidobacterium* when compared to dosing bi-weekly or weekly (52).

A lower relative abundance of *Bifidobacterium, Lactobacillus, and Veilonella*, and a higher relative abundance of *Escherichia*, were observed at day 28 and 4 months of age among infants treated with broad-spectrum compared to narrow-spectrum antibiotic regimens. Reduced abundance of protective anaerobe commensals and higher abundance of *Enterobacteriaceae* after antibiotic exposure has also previously been reported (53, 54). When comparing presence and absence of antibiotic exposure after the first week of life, no differences in diversity or taxonomic composition were found. Previous studies on alpha diversity and influence of antibiotic treatment have shown inconsistent results (55). However, infants who were most heavily exposed to antibiotic treatment in our study were also supplemented with probiotics. In animals, probiotics may alleviate the potential loss of microbial diversity created by antibiotic treatment (56). This may explain why PEP-infants, exposed to massive antibiotic pressure, did not have reduced microbial gut diversity compared to other groups. Thus, probiotic supplementation may offer a protective effect partly compensating harmful effects of antibiotics in preterm infants. However, the early low number of taxa in preterm infant stools places constraints on interpreting diversity changes as diversity in a non-complex population may reflect changes in only one taxon.

In line with others, we found that the gut antibiotic resistome of preterm and term infants is established early, independent of antibiotic exposure (21, 57–59). We detected significant higher abundance of ARGs in infants receiving broad-spectrum antibiotics compared to narrow-spectrum regimens. Gibson and co-workers also showed that broad-spectrum antibiotic therapy in preterm infants, was associated with enrichment of specific ARGs (21). We aimed to investigate how probiotic supplementation can influence the gut antibiotic resistome. Overall, there were no differences in distribution of ARG-classes or abundance of ARGs at 28 days and 4 months of age between PEP-infants, exposed to massive antibiotic therapy, and the two other groups with limited or no antibiotic exposure. One possible mechanism for this finding is that probiotic bacteria can produce bacteriocins that improve mucosal integrity and thereby reduces the pathogenic bacterial population and antibiotic resistance (60).

### Strengths and limitations

At the time of this study, probiotic supplementation to extremely preterm infants was considered “standard of care” in Norway. We were therefore beyond equipoise to perform a randomized study comparing probiotic to no probiotic supplementation in this population. The NPVP-infant group has limitations as a control group due to maturational differences and the difference in antibiotic exposure compared to the PEP-infants. However, more antibiotic exposure in the PEP-infants would most likely have led to less diversity and higher abundance of ARGs. Still, we found few differences between the two preterm groups at 28 days and 4 months of age, suggesting a protective effect of probiotics in the PEP-infant group. The gut microbiota composition of preterm infants may differ between hospitals (61), but our multi-center approach intended to average local differences and strengthen generalizability. Infants harbor a much lower gut microbial diversity compared to adults. Any variation in the gut microbiota composition caused by storage may thus theoretically have a proportionally greater effect on the composition (25). We chose a standardized sampling technique in order to avoid potential biases due to freezing of samples at different time points and temperature variation during transport to the laboratory. However, in the most immature infants the DNA content in the early fecal samples was very low, and we were only able to obtain sequence data from 20/31 samples at 1 week of age.

## Conclusion

Probiotic-supplemented extremely preterm (PEP) infants had a high relative abundance of *Bifidobacterium* at 1 week of age, only few days after start of probiotic supplementation. PEP-infants were also exposed to much more antibiotics, but overall microbial diversity and resistome was not different than in more mature infants at 4 weeks and 4 months. We speculate that probiotic supplementation may induce colonization resistance and thereby partly alleviate harmful effects of antibiotics on gut microbiota composition and the antibiotic resistome development.

## Data availability

The raw data supporting the conclusion of this manuscript will be made available by the authors, without undue reservation, to any qualified researcher.

## Author contributions

EE organized all phases of the study, analyzed data, wrote the first version of the manuscript, and revised the manuscript. She had full access to all of the data in the study and takes responsibility for the integrity of the data and the accuracy of the data analysis. TP, JA, SR, RS, and BN were responsible for inclusion of patients at participating centers, data retrieval, and revised the manuscript. JC, EH, and NW took part in study design, were responsible for microbiological (JC) and bioinformatic (EH, NW) analyses and revised the manuscript. CK conceptualized and designed the study, directed all phases of the study, and revised the final manuscript. He had full access to all of the data in the study and takes responsibility for the integrity of the data and the accuracy of the data analysis. All authors approved the final manuscript as submitted and agree to be accountable for all aspects of the work.

### Conflict of interest statement

The authors declare that the research was conducted in the absence of any commercial or financial relationships that could be construed as a potential conflict of interest.

## Abbreviations

ARG: Antibiotic resistance genes
CARD: Comprehensive antibiotic resistance database
CFU: Colony forming units
FDR: False discovery rate
FTC: Full-term control
NEC: Necrotizing enterocolitis
NICU: Neonatal intensive care unit
NMDS: Non-metrical multidimensional scaling
NVPVP: Non-probiotic very preterm
PEP: Probiotic extremely preterm.
